# A 2-Year Longitudinal Study of Normal Cone Photoreceptor Density

**DOI:** 10.1167/iovs.18-25904

**Published:** 2019-04

**Authors:** Kevin Jackson, Grace K. Vergilio, Robert F. Cooper, Gui-Shuang Ying, Jessica I. W. Morgan

**Affiliations:** 1 Scheie Eye Institute, Department of Ophthalmology, Perelman School of Medicine, University of Pennsylvania, Philadelphia, Pennsylvania, United States; 2 Center for Advanced Retinal and Ocular Therapeutics, Department of Ophthalmology, University of Pennsylvania, Philadelphia, Pennsylvania, United States; 3 Department of Psychology, University of Pennsylvania, Philadelphia, Pennsylvania, United States

**Keywords:** adaptive optics, cones, photoreceptors, scanning laser ophthalmoscopy

## Abstract

**Purpose:**

Despite the potential for adaptive optics scanning light ophthalmoscopy (AOSLO) to quantify retinal disease progression at the cellular level, there remain few longitudinal studies investigating changes in cone density as a measure of disease progression. Here, we undertook a prospective, longitudinal study to investigate the variability of cone density measurements in normal subjects during a 2-year period.

**Methods:**

Fourteen eyes of nine subjects with no known ocular pathology were imaged both at a baseline and a 2-year follow-up visit by using confocal AOSLO at five retinal locations. Two-year affine-registered images were created to minimize the effects of intraframe distortions. Regions of interest were cropped from baseline, 2-year manually aligned, and 2-year affine-registered images. Cones were identified (graded masked) and cone density was extracted.

**Results:**

Mean baseline cone density (cones/mm^2^) was 87,300, 62,200, 45,500, 28,700, and 18,200 at 190, 350, 500, 900, and 1500 μm, respectively. The mean difference (± standard deviation [SD]) in cone density from baseline to 2-year affine-registered images was 1400 (1700), 100 (1800), 300 (800), 400 (800), and 1000 (2400) cones/mm^2^ at the same locations. The mean difference in cone density during the 2-year period was lower for affine-registered images than manually aligned images.

**Conclusions:**

There was no meaningful change in normal cone density during a 2-year period. Intervisit variability in cone density measurements decreased when intraframe distortions between time points were minimized. This variability must be considered when planning prospective longitudinal clinical trials using changes in cone density as an outcome measure for assessing retinal disease progression.

Confocal adaptive optics scanning light ophthalmoscopy (AOSLO)^1^ has been used to observe the normal cone mosaic^2^–^4^ as well as the abnormal cone mosaics present in retinal diseases including AMD,^5^,^6^ retinitis pigmentosa,^7^–^9^ choroideremia,^10^,^11^ achromatopsia,^12^ central serous chorioretinopathy,^13^ and others.^14^ With the ability to obtain images with cellular resolution in vivo, AOSLO along with other adaptive optics ophthalmoscopy techniques has overcome two major limitations of histology: the ability to assess cell structure cross-sectionally in patients before death (thereby increasing the number of patients available to study), and the ability to follow up these same patients over time. Indeed, numerous investigators have proposed AOSLO imaging as a potential method for tracking individual cell survival in disease and its treatment, and there are several “proof-of-concept” reports where longitudinal AOSLO imaging has demonstrated the ability to track retinal structure at the cellular level.^2^,^15^–^17^ As a result, interest in using cone mosaic metrics as an outcome measure for assessing the safety and efficacy of experimental interventions in clinical trials is increasing. However, for cone density (or any other AOSLO-based metric) to be most effective as an outcome measure, investigators must first understand how cone density changes throughout the natural progression of a disease in comparison with how cone density changes in the absence of disease (i.e., aging). Further, investigators must understand the variability of longitudinal cone density measurements, as this will impact the sensitivity with which cone density can be used in clinical trials to detect changes in the photoreceptor mosaic. Here, we take a step forward in this process by undertaking a prospective, longitudinal study to investigate cone density in normal subjects during a 2-year period.

## Methods

The research study was approved by the institutional review board at the University of Pennsylvania and followed the tenets of the Declaration of Helsinki. Following explanation of the study requirements and potential risks, subjects gave informed consent and voluntarily enrolled in the study.

Fourteen eyes with no known retinal pathology were included in this study. Axial lengths for each eye were obtained by using an IOL Master (Carl Zeiss Meditec, Dublin, CA, USA) at each study visit and were used to calculate the scale for AOSLO images at each time point. Best corrected visual acuity was measured at each study visit for each eye independently. Subjects were imaged at baseline and again at 2 years (study window, 22–26 months). For each imaging session, subjects' eyes were dilated with phenylephrine hydrochloride (2.5%) and tropicamide (1%), and a dental impression “bite bar” was used to align the subject to the AOSLO.

### Imaging the Photoreceptor Mosaic

The AOSLO used in this study has been previously described.^18^ Aberration correction was completed by using a 97 actuator deformable mirror (Alpao, Montbonnot, France), wavefront sensing was performed by using a superluminescent diode centered at 848 nm (Superlum, Cork, Ireland), and imaging was done by using a superluminescent diode centered at 795 nm (Superlum). One photomultiplier tube (Hamamatsu Corporation, Hamamatsu City, Japan) was used to acquire a confocal reflectance video of the retina from light passing through a confocal pinhole placed optically conjugate with the photoreceptor layer.

Subjects were instructed to fixate (using the imaged eye) as steadily as possible at a target while the AOSLO image sequences were acquired. High-resolution AOSLO images were obtained around the fovea and along the temporal meridian out to approximately 1500 μm at baseline and at 2 years. An operator manually selected one high-quality frame from each image sequence. This frame was used as the reference frame for a custom strip-registration software^19^ that was used to first dewarp the effects of the sinusoidal scan on the confocal videos and then to register and average 50 frames of each AOSLO video together to increase the signal to noise ratio of the final averaged image.

### Analyzing the Photoreceptor Mosaic

**Figure 1:**
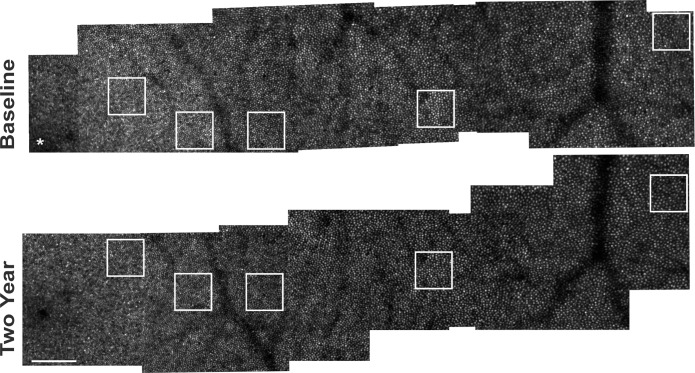
Baseline and 2-year confocal AOSLO montages obtained along the temporal meridian for subject 11002. White boxes outline the ROIs selected for cone density analysis at 190, 350, 500, 900, and 1500 μm. White asterisk (*) denotes the location of the fovea. Scale bar: 100 μm.

The registered, averaged images were automatically montaged by using a previously described custom AOSLO retinal image montaging algorithm (Fig. 1).^20^ AOSLO montages were generated for the baseline and 2-year follow-up study visits individually. The 2-year follow-up montages were scaled to the baseline montage (Adobe Photoshop; Adobe Inc., San Jose, CA, USA) by using the difference between the theoretical image scales at each individual study session (based on the subject's axial length and an AOSLO image of a Ronchi ruling with known line spacing). For each eye, the 2-year AOSLO montages were manually aligned to the baseline AOSLO montages. From the montages, an operator used a custom MATLAB (MathWorks, Inc., Natick, MA, USA) software to extract selected retinal regions of interest (ROI) at five retinal locations, namely, 190, 350, 500, 900, and 1500 μm from the fovea, with eccentricity being calculated as the distance from the fovea to the center of the ROI. If the ROI was found to have significant image quality defects or overlap with blood vessels, the ROI was minimally displaced (within 10% of the designated retinal location except for one image at 1500 μm, which was moved 15%) to obtain an acceptable image. Once the ROI locations were identified on the montage, an operator verified the local alignment between the baseline and 2-year images within the montages. Using both translational and rotational movement only, the 2-year follow-up image (approximately 1° square in size) was manually adjusted with Adobe Photoshop to obtain the best alignment with the baseline image at the retinal location corresponding to the ROI. Finally, 85-μm-per-side square ROIs were extracted from the baseline and 2-year follow-up manually aligned images.

Baseline and 2-year follow-up images were manually aligned as best as possible; however, accurate cell-by-cell alignment was cumbersome and difficult. Residual distortions (caused by fixational eye motion during image acquisition) in the registered images were different between the baseline and 2-year follow-up images, sometimes resulting in misalignments even over the small ROI size. To determine the effect of different intraframe distortions longitudinally, a second strip-registration process was conducted. First, the image intensity in both the baseline and 2-year follow-up processed images (approximately 1° square in size) was normalized by using contrast-limited adaptive histogram equalization (CLAHE) in Fiji.^21^ The CLAHE-normalized 2-year follow-up image was then registered (using the strip-registration software described above) to the baseline image (which was used as the reference). Using Fiji,^21^ an affine transformation was performed on the strip-registered 2-year follow-up image to remove shear, torsional, and scale distortions as best as possible. Finally, an 85-μm-per-side square ROI that aligned with the baseline ROI at each location was manually extracted from the 2-year follow-up affine registered image.

Square ROIs, 85 μm per side, were cropped from the baseline, 2-year follow-up manually aligned, and 2-year follow-up affine-registered images, resulting in a total of 210 ROIs. Semiautomated custom software was used to identify cones in each ROI by a single experienced grader (JIWM). Automated cone selections were made by finding local maxima in image brightness, based on previously described algorithms.^22^,^23^ The grader was able to manually add or remove automatically identified cones and could adjust the brightness and contrast of the image on both a log or linear scale. Cone locations were identified in baseline and 2-year follow-up manually aligned images first (graded while masked to subject, eye, retinal location, and time point), followed by analysis of the 2-year affine-registered images at a later date (graded while masked to subject, eye, and retinal location). Cone locations were used to determine bound cone density over each ROI. To calculate bound cone density, each identified cone location served as a Voronoi cell center. Voronoi cells whose boundaries extended beyond the ROI were termed “unbound” and excluded from further analysis. Bound cone density was then calculated by dividing the total number of bound Voronoi cells in an ROI by the total bound Voronoi area within the ROI.^24^

### Statistical Analysis

We calculated the mean and SD of cone density measured at each of five eccentricities (190, 350, 500, 900, and 1500 μm) at baseline and at 2 years in both manually aligned and affine-registered images. The mean (and SD) difference between baseline and 2-year cone density was assessed at each eccentricity. Limits of agreement between baseline and 2-year follow-up data were calculated as mean ± 1.96 * SD of their difference. Statistical significance for differences between baseline and 2-year cone densities was assessed through linear regression analysis by using a mixed effects model^25^ to account for intereye correlation, since both eyes of some subjects were included for the study. All statistical analyses were performed with SAS v9.4 (SAS, Cary, NC, USA) and *P* < 0.05 was considered to be statistically significant.

## Results

Subject demographics for this study are presented in Table 1. Fourteen eyes of nine subjects aged 18 to 54 years at baseline (mean ± SD: 28 ± 11 years) were included in the study. Best corrected visual acuity was 20/20 or better for all eyes included in the study at the time of both the baseline and 2-year imaging session. Baseline axial lengths varied from 23.58 to 27.48 mm with mean (SD) of 25.26 (1.67) mm. Axial lengths at the 2-year follow-up visit varied from 23.56 to 27.52 mm with mean (SD) of 25.30 (1.67). The mean (SD) change in axial length between baseline and 2-year study visits was 0.04 (0.04) mm.

**Table 1:** Subject Characteristics

**Subject ID**	**Age at Baseline, y**	**Sex**	**Baseline Axial Length, mm**		**Baseline BCVA**	
			**OD**	**OS**	**OD**	**OS**
11002	33	F	*	25.78	*	20/20
11015	23	F	27.48	*	20/16	*
11018	18	M	27.06	27.17	20/16	20/16
11028	28	M	24.01	23.92	20/12.5	20/12.5
11037	23	M	25.94	26.01	20/16	20/12.5
11038	20	M	24.41	24.17	20/20	20/20
11040	28	F	24.21	23.58	20/16	20/12.5
11044	26	M	24.72	*	20/12.5	*
11046	54	M	25.2	*	20/16	*

BCVA, best corrected visual acuity.

* Data from this eye was not acquired with AOSLO imaging at baseline.

**Figure 2:**
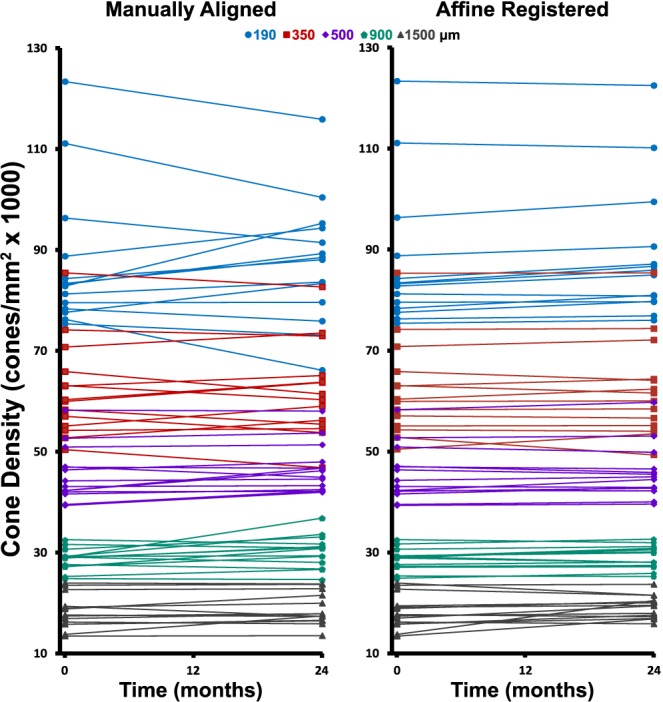
Cone densities measured at baseline and at 2 years were manually aligned (left column) and affine registered (right column) in 14 normal eyes from 9 subjects.

**Figure 3:**
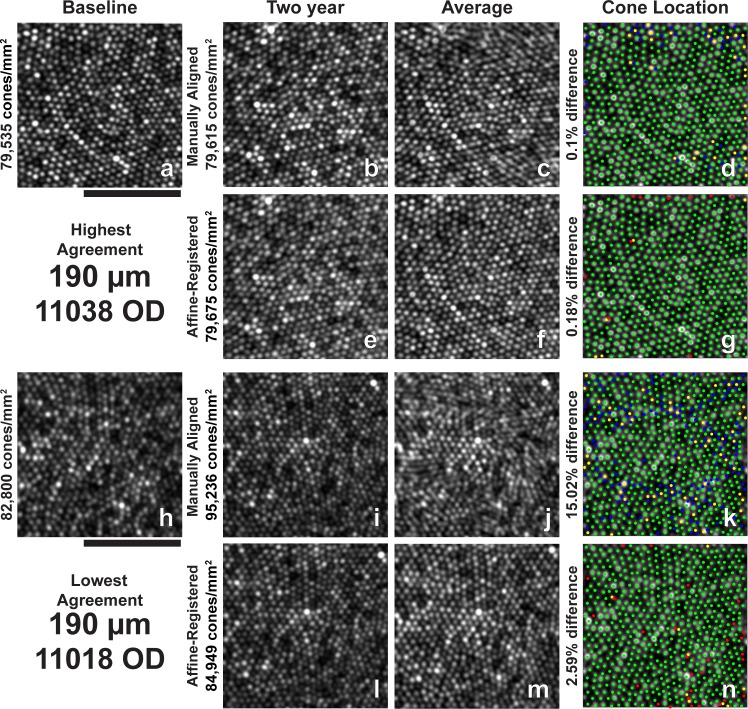
Images and all cone locations (bound and unbound) identified at 190-μm ROI with the highest and lowest absolute agreement in bound cone density between baseline and 2-year follow-up images. (a) Baseline image for highest agreement. (b) Two-year manually aligned image for highest agreement. (c) Average of baseline and 2-year manually aligned images in (a) and (b). (d) Cone locations identified in both the baseline and 2-year manually aligned images (green), only the baseline image (yellow), and only the 2-year manually aligned image (blue) overlaid on the baseline image (a). (e) Two-year affine-registered image for highest agreement. (f) Average of baseline and 2-year affine-registered images in (a) and (e). (g) Cone locations identified in both the baseline and 2-year affine-registered images (green), only the baseline image (yellow), and only the 2-year affine-registered image (red) overlaid on the baseline image (a). (h) Baseline image for lowest agreement. (i) Two-year manually aligned image for lowest agreement. (j) Average of baseline and 2-year manually aligned images in (h) and (i). (k) Cone locations identified in both the baseline and 2-year manually aligned images (green), only the baseline image (yellow), and only the 2-year manually aligned image (blue) overlaid on the baseline image (h). (l) Two-year affine-registered image for lowest agreement. (m) Average of baseline and 2-year affine-registered images in (h) and (l). (n) Cone locations identified in both the baseline and 2-year affine-registered images (green), only the baseline image (yellow), and only the 2-year affine-registered image (red) overlaid on the baseline image (h). Misalignments and noncommon distortions between baseline and 2-year manually aligned images can be observed as smudged or blurred cones in the average image (c) and (j). Affine alignment improves the alignment and compensates for noncommon distortions between the time points, resulting in a less blurred average image (f, m). All images are square 85 μm. Scale bar: 50 μm.

**Figure 4:**
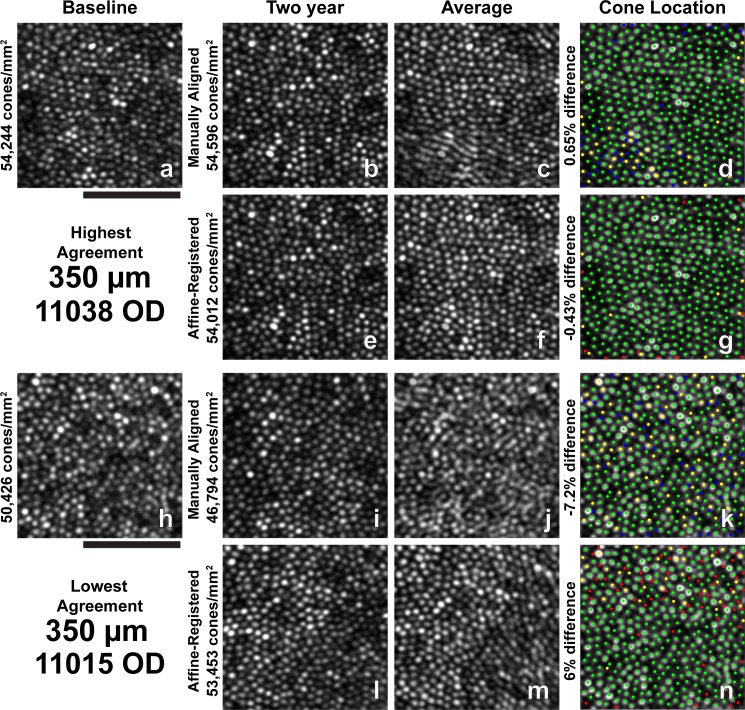
Images and all identified cone locations at 350-μm ROI with the highest and lowest agreement in bound cone density between baseline and 2-year follow-up images. (a–n) Same as in Figure 3. Scale bar: 50 μm.

**Figure 5:**
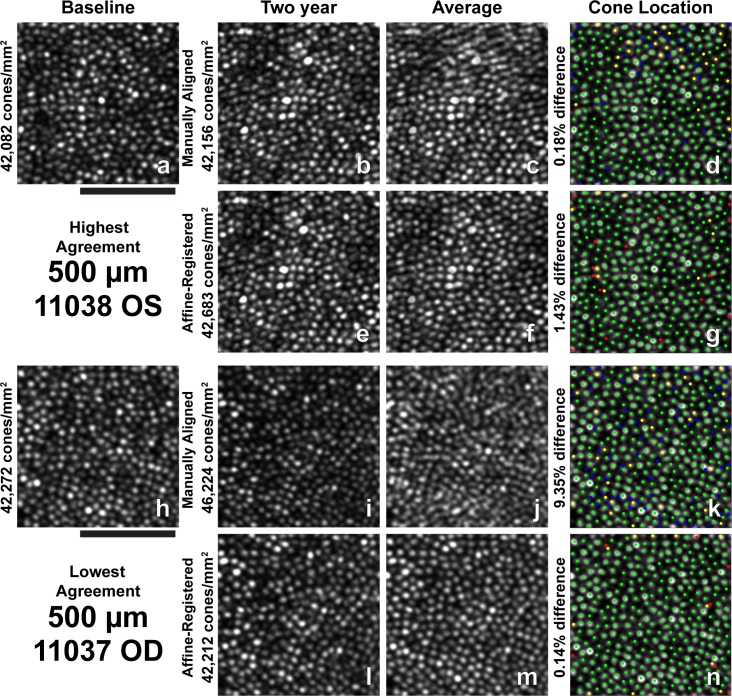
Images and all identified cone locations at 500-μm ROI with the highest and lowest agreement in bound cone density between baseline and 2-year follow-up images. (a–n) Same as in Figure 3. Scale bar: 50 μm.

**Figure 6:**
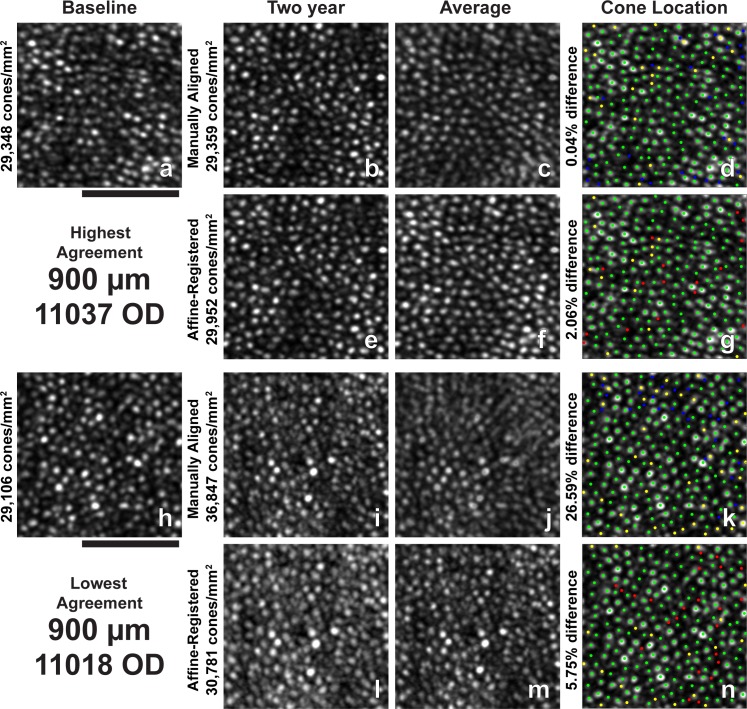
Images and all identified cone locations at 900-μm ROI with the highest and lowest agreement in bound cone density between baseline and 2-year follow-up images. (a–n) Same as in Figure 3. Scale bar: 50 μm.

**Figure 7:**
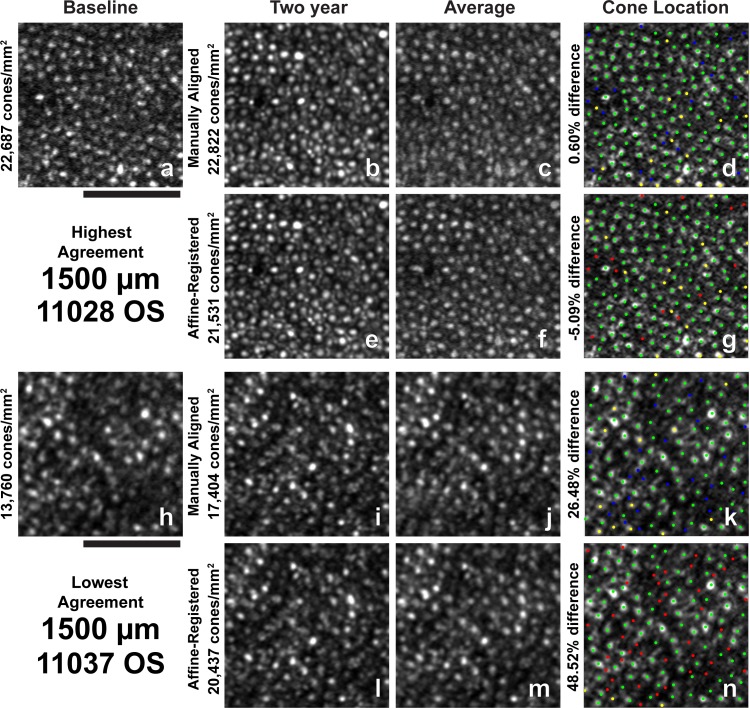
Images and all identified cone locations at 1500-μm ROI with the highest and lowest agreement in bound cone density between baseline and 2-year manually aligned images. (a–n) Same as in Figure 3. Scale bar: 50 μm.

Mean baseline cone density for the normal subjects included in this study was 87,300, 62,200, 45,500, 28,700, and 18,200 cones/mm^2^ at 190, 350, 500, 900, and 1500 μm, respectively (Table 2). Baseline measurements of cone density showed high intersubject variability quantified by the large SDs (noted in Table 2) at all parafoveal retinal eccentricities included in the study. Mean cone density from the 2-year manually aligned and 2-year affine-registered data is displayed in Table 2 along with the difference in cone density between the baseline and 2-year data. Cone density was stable during the 2-year period for most locations, with 2-year affine-registered cone densities exhibiting smaller differences from baseline than 2-year manually aligned cone densities (Fig. 2). The difference between baseline and 2-year manually aligned paired cone densities ranged in absolute magnitude from 0.04% to 26.59% of their baseline value (Figs. 3–7). There was no statistically significant difference in cone density measurements between baseline and 2-year manually aligned images at 190-, 350-, 900-, or 1500-μm eccentricities. There was a statistically significant increase in cone density of 1000 ± 1500 cones/mm^2^ at the 500-μm eccentricity (Table 2).

**Table 2:** Mean Cone Density and Difference From Baseline Versus Retinal Eccentricity

	**Retinal Eccentricity**				
	**190 μm**	**350 μm**	**500 μm**	**900 μm**	**1500 μm**
Mean density, cones/mm^2^ × 1000					
Baseline	87.3 ± 17.6	62.2 ± 11.2	45.5 ± 6.0	28.7 ± 2.3	18.2 ± 4.2
Two-year MA	87.5 ± 14.2	62.1 ± 11.1	46.6 ± 6.0	30.3 ± 3.7	18.7 ± 3.9
Two-year AR	88.7 ± 16.5	62.2 ± 11.5	45.7 ± 6.3	29.1 ± 2.3	19.2 ± 2.6
Difference from baseline, cones/mm^2^ × 1000					
Two-year MA	0.2 ± 7.6	−0.1 ± 2.8	1.1 ± 1.5	1.6 ± 2.7	0.5 ± 1.3
*P* values	0.93	0.94	0.03*	0.11	0.17
Limit of agreement	−14.7 to 15.1	−5.6 to 5.4	−1.8 to 4.1	−3.7 to 6.9	−2.0 to 3.1
Two-year AR	1.4 ± 1.7	0.1 ± 1.8	0.3 ± 0.8	0.4 ± 0.8	1.0 ± 2.4
*P* values	0.01*	0.63	0.34	0.14	0.15
Limit of agreement	−1.9 to 4.7	−3.5 to 3.6	−1.3 to 1.8	−1.2 to 1.9	−3.7 to 5.7

Data are shown as mean ± SD. AR, affine registered; MA, manually aligned.

* *P* < 0.05.

**Figure 8:**
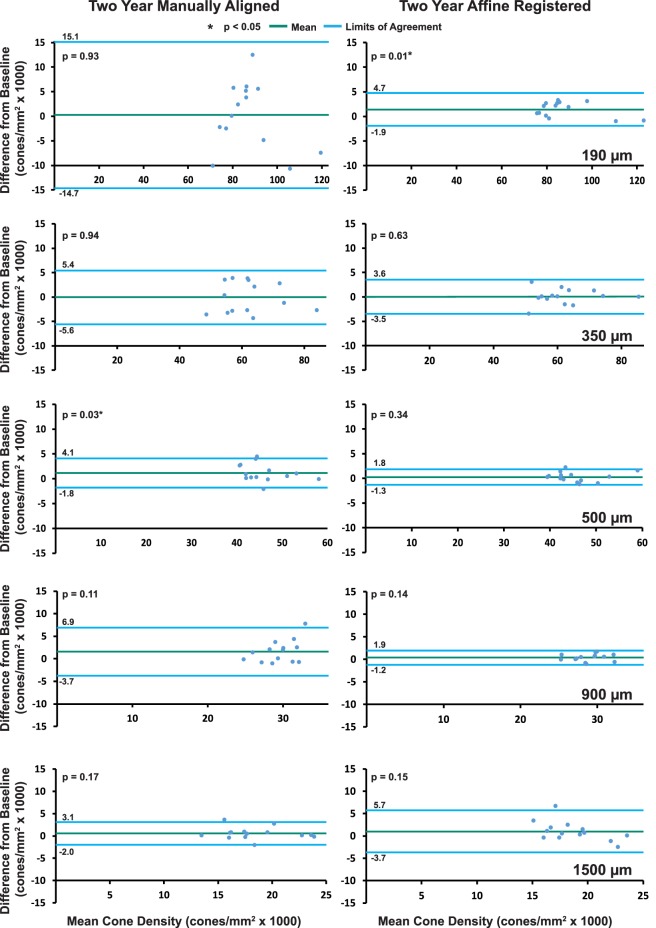
Bland-Altman plots between 2-year and baseline cone density measurements for each ROI eccentricity (Left column: 2-year manually aligned; Right column: 2-year affine registered). Mean and limits of agreement in cone density from baseline are shown by green and blue lines, respectively. A significant increase in cone density was observed for the 500-μm manually aligned and the 190-μm affine-registered locations. The limits of agreement decreased in affine-registered images in comparison to manually registered images at all eccentricities except for 1500 μm.

Two-year follow-up affine-registered images aligned better to baseline images than the manually aligned images. Following affine registration, it was determined that approximately 27% (19/70) of the manually aligned images had a translational misalignment in addition to interimage distortions in the manually aligned and baseline images. Further, baseline and manually aligned images showed different intraframe distortions, which precluded cone-by-cone alignment over the full ROI in some cases. The different intraframe distortions can be observed as blurred cones in average images of the baseline and 2-year manually aligned images (Figs. 3c, 3j, 4c, 4j, 5c, 5j, 6c, 6j, 7c, 7j). Affine registration of the 2-year follow-up images to the baseline images corrected the translational misalignment and greatly reduced, but did not eliminate, the differences in intraframe image distortions (Figs. 3f, 3m, 4f, 4m, 5f, 5m, 6f, 6m, 7f, 7m). Affine-registered cone densities at 2-year follow-up visits were not significantly different from baseline cone density measurements at 350, 500, 900, or 1500 μm. There was a statistically significant increase in cone density of 1400 ± 1700 cones/mm^2^ at the 190-μm eccentricity (Table 2). The limits of agreement decreased in the 2-year affine-registered cone densities at 190, 350, 500, and 900 μm in comparison with the 2-year manually aligned cone densities, but increased at 1500 μm (Fig. 8; Table 2).

## Discussion

To date, most AOSLO imaging and related analysis have involved comparing cone densities in normal and diseased retinas through cross-sectional studies. Although many investigators have touted the capability of AOSLO imaging to assess retinal structure longitudinally at the cellular scale, there are very few studies that have quantified cellular structure over time.^26^,^27^ Before AOSLO imaging can become a useful outcome measure for assessing retinal disease progression and treatment response, investigators must first understand if and how the retina changes in normal subjects over the same period. In this study, we assessed cone density in normal subjects during a 2-year period, which is the time point associated with the primary endpoint for many therapeutic clinical trials.^28^,^29^ Thus, this study represents a necessary step for incorporating AOSLO metrics into longitudinal studies of disease progression and for using AOSLO metrics as endpoints for clinical trials assessing treatment safety and efficacy.

### Normal Cone Density During the 2-Year Period

We conclude that there is no meaningful change in normal cone density during a 2-year period. First, we would expect on a priori grounds for cone density to either remain constant or to decrease during the 2-year period, and we found no statistically significant decrease in cone density at any ROI eccentricity in our study. Second, although we did find a nominally statistically significant increase in cone density at one location under each 2-year image registration scheme, we do not view this increase as persuasive evidence of meaningful retinal change. Cross-sectional studies^2^,^3^,^30^ have provided evidence that cone density will either remain stable or decrease throughout adulthood. Using histologic methods and intersubject comparisons, Curcio et al.^30^ have found that cone density is stable during adulthood. Alternatively, using AOSLO imaging, Song et al.^2^ have found that cone density is statistically lower in older (50–65 years old) than younger (20–35 years old) individuals within 0.45 mm of the fovea, while there is no statistically significant difference at eccentricities greater than 0.45 mm from the fovea. Park et al.^3^ have found a slightly negative correlation between cone density and age, though this correlation is not statistically significant. While our study found no loss of cones during a 2-year period, we are unable to adequately address the outstanding question of whether and how much cone loss occurs over adulthood, both because of the young age of our study population and the relatively short follow-up time. Longer follow-up time windows could resolve this apparent discrepancy regarding the rate of cone loss throughout adulthood. However, for shorter periods, 2 years or less, we conclude that cone density in normal subjects remains stable.

### Effect of Intraframe Distortions

Significant reduction in the variation of cone density measurements was found after reducing intraframe distortions and misalignments between imaging sessions. These results are consistent with the findings of Garrioch et al.^23^ and Cooper et al.^31^ who have detailed the presence of different intraframe distortions between images and shown better repeatability in cone density measurements with minimization of intraframe distortions. Although the intraframe distortions are not readily apparent when viewing an individual ROI, they are apparent when viewing multiple images together (Figs. 3–7, average of baseline and manually aligned images). These distortions (compressions, expansions, and torsions) can lead to the addition (or removal) of a row of cones in the 2-year image in comparison with the baseline image, thus resulting in more (or less) bound cones being included in the ROI.

Affine-registration of 2-year images to baseline images reduced the variability observed in cone density measurements during the 2-year period by attempting to minimize the difference in intraframe distortions between the images acquired at different time points. This mostly corrected the addition or removal of a row of cones in the ROI at the later time point in comparison with baseline (Figs. 3–7, average of baseline and affine-registered images), although some differences in distortions remain between the two time points. This effect is likely eccentricity dependent, as the same spatial distortion will cause a greater effect on bound cone density when cone spacing is low (low eccentricities) compared to high (high eccentricities).

It is important to note that the affine registration process did not create “distortion-less” AOSLO retinal images. Instead, the processing distorts the 2-year manually aligned images so that they best match the baseline images (and the distortions included within them). This process is mostly applicable when there is no change in the cone photoreceptor mosaic, as was generally found in the 2-year manually aligned data. Therefore, although this process works well in normal controls over the relatively short timeframe here, its use may not be applicable when true retinal change occurs between time points. For example, retinal changes in patients with ocular disease could be too large to allow accurate distortion matching between time points. Conversely, small retinal changes could be erroneously removed by forcing images to match each other. It is worth noting that cone density measurements could still be used to evaluate disease progression without correcting for intraframe distortions; however, the increased variability in measurements caused by intraframe distortions will require a larger change in cone density from baseline before a statistically significant “true retinal change” can be determined. Thus, we expect the best results for measuring true retinal change in short periods will come when intraframe distortions are minimized at each time point individually before images from different time points are aligned. As a result, there remains a critical need for a method that can obtain truly “distortionless” AOSLO images.

In addition to minimizing the effect of intraframe distortion differences with time, the affine registration process revealed previously unknown errors in the manual alignments. Despite our best attempts to attain cell-by-cell alignment manually (with significant operator time dedicated solely to this task), approximately 27% (19/70) of the ROIs had translation misalignments greater than the width of a single cone. Correcting the misalignments likely contributed in part to the reduced limits of agreement observed for affine-registered images versus manually registered images. This effect is most predominantly observed at the 190-μm location, where misalignments would have a greater effect on cone density differences owing to the higher gradient in cone density with retinal eccentricity at this location. We attribute the misalignments between images to the fact that the cone mosaic is highly regular, with a mostly triangular packing arrangement and highly heterogeneous reflectance pattern. The reflectance of cones is known to change over time,^32^ thereby making the alignments more difficult, since matching cone reflectance intensities will not result in an accurate alignment. Thus, automated alignment algorithms, such as that detailed by Chen et al.^20^ or Davidson et al.^33^ but adapted for longitudinal image alignment, will be required before longitudinal AOSLO imaging is routinely used. Split detection AOSLO imaging,^34^ which is immune to the variable intensities observed in waveguided cone reflectance, may also help with longitudinal montaging, though split detection imaging was not available for our baseline study visits.

### Repeatability of AOSLO Measurements of Cone Density and Other Sources of Variability in Longitudinal Cone Density Measurements

Beyond intraframe distortions, image misalignments, and true retinal change, numerous other factors can contribute to variability in longitudinal cone density measurements. For example, differences in overall image quality, the visibility of individual cone reflectance, and scale between time points could all contribute to the variability observed. Cone reflectance is known to change over time and can contribute to variable visibility of cones, potentially resulting in different cone identifications (Figs. 7k, 7n). However, post hoc inspection of all ROIs including the 190-μm and 500-μm locations did not reveal any systematic differences in image quality at baseline in comparison with 2 years, and we corrected for theoretical scaling differences between time points.

In addition to the factors listed above, longitudinal cone density measurements also will be limited by the repeatability of individual cone density measurements. Therefore, to make a conclusion about how the retina changes in normal subjects over time, we must also understand the precision of our measurement technique. Previous studies have examined the repeatability and reliability of cone density measurements over a single imaging session. At 190 μm, Garrioch et al.^23^ have found a repeatability of 2.7% or 2000 cones/mm^2^ for cone density measurements obtained during the same imaging session. For reference, the mean difference in cone density at the statistically significant ROIs of 190 μm and 500 μm in our study were 1400 and 1100 cones/mm^2^ (1.6% and 2.4%), respectively. Morgan et al.^35^ have found that intergrader agreement in confocal cone density measurements decreases with eccentricity, where this decrease is attributed in part to the visibility of rods in confocal AOSLO images. Likewise, the higher limits of agreement observed at the 1500-μm ROI location may be caused in part by the presence of rods in the images (we cannot exclude the possibility that some rods were misidentified as cones at higher eccentricities), thus leading to reduced agreement in cone identifications over time, though the effect of eccentricity on the repeatability of cone density measurements is still unknown. Morgan et al.^35^ also have found that cone density measurements made from split detection AOSLO images^34^ show higher agreement between graders in comparison to location-matched confocal images. Thus, using split detection imaging may reduce the variability found in the present study at the higher eccentricities (in particular at 1500 μm); however, current split detection AOSLO imaging does not reveal the cone inner segment mosaic at retinal locations close to the fovea in most normal subjects. In the current study, split detection AOSLO imaging was not available at the time of our baseline measurements; however, future studies certainly will benefit by using multimodal approaches to observe and identify cone locations over time. Regardless, the repeatability and reliability studies from Garrioch et al.^23^ and Morgan et al.^35^ further support our conclusion that the two statistically significant locations that showed a relatively small increase in cone density in the present study do not actually represent meaningful changes during the 2-year period.

### Future Directions

Gene, small molecule, stem cell, and optogenetic therapies^36^–^40^ for blinding disease have developed in parallel with AOSLO. Each of these therapies operates on the level of individual cells, yet there remains a lack of outcome measures capable of assessing disease progression and treatment response at the cellular level. AOSLO has the potential to fill this void, since one of its main advantages is that the same cells can be followed over time. Here, we showed no significant decrease in cone density during a 2-year period in predominantly young subjects with normal vision. This information will become critical as cone density measurements are increasingly incorporated into longitudinal studies and clinical trials as a sensitive cellular-level outcome measure for assessing disease progression and treatment response.
